# Fengycins, Cyclic Lipopeptides from Marine Bacillus subtilis Strains, Kill the Plant-Pathogenic Fungus Magnaporthe grisea by Inducing Reactive Oxygen Species Production and Chromatin Condensation

**DOI:** 10.1128/AEM.00445-18

**Published:** 2018-08-31

**Authors:** Linlin Zhang, Chaomin Sun

**Affiliations:** a CAS Key Laboratory of Experimental Marine Biology, Institute of Oceanology, Chinese Academy of Sciences, Qingdao, China; b Laboratory for Marine Biology and Biotechnology, Qingdao National Laboratory for Marine Science and Technology, Qingdao, China; c University of Chinese Academy of Sciences, Beijing, China; d Center for Ocean Mega-Science, Chinese Academy of Sciences, Qingdao, China; University of Toronto

**Keywords:** Bacillus species, lipopeptide, chromatin condensation, fengycin, proteomic analyses, reactive oxygen species production

## Abstract

Rice blast caused by the phytopathogen Magnaporthe grisea poses a serious threat to global food security and is difficult to control. Bacillus species have been extensively explored for the biological control of many fungal diseases. In the present study, the marine bacterium Bacillus subtilis BS155 showed a strong antifungal activity against M. grisea. The active metabolites were isolated and identified as cyclic lipopeptides (CLPs) of the fengycin family, named fengycin BS155, by the combination of high-performance liquid chromatography (HPLC) and electrospray ionization mass spectrometry (ESI-MS) and tandem mass spectrometry (ESI-MS/MS). Analyses using scanning and transmission electron microscopy revealed that fengycin BS155 caused morphological changes in the plasma membrane and cell wall of M. grisea hyphae. Using comparative proteomic and biochemical assays, fengycin BS155 was demonstrated to reduce the mitochondrial membrane potential (MMP), induce bursts of reactive oxygen species (ROS), and downregulate the expression level of ROS-scavenging enzymes. Simultaneously, fengycin BS155 caused chromatin condensation in fungal hyphal cells, which led to the upregulation of DNA repair-related protein expression and the cleavage of poly(ADP-ribose) polymerase (PARP). Altogether, our results indicate that fengycin BS155 acts by inducing membrane damage and dysfunction of organelles, disrupting MMP, oxidative stress, and chromatin condensation, resulting in M. grisea hyphal cell death. Therefore, fengycin BS155 and its parent bacterium are very promising candidates for the biological control of M. grisea and the associated rice blast and should be further investigated as such.

**IMPORTANCE** Rice (Oryza sativa L.) is the most important crop and a primary food source for more than half of the world's population. Notably, scientists in China have developed several types of rice that can be grown in seawater, avoiding the use of precious freshwater resources and potentially creating enough food for 200 million people. The plant-affecting fungus Magnaporthe grisea is the causal agent of rice blast disease, and biological rather than chemical control of this threatening disease is highly desirable. In this work, we discovered fengycin BS155, a cyclic lipopeptide material produced by the marine bacterium Bacillus subtilis BS155, which showed strong activity against M. grisea. Our results elucidate the mechanism of fengycin BS155-mediated M. grisea growth inhibition and highlight the potential of B. subtilis BS155 as a biocontrol agent against M. grisea in rice cultivation under both fresh- and saltwater conditions.

## Abstract (precis)

Rice (Oryza sativa L.) is the most important crop and a primary food source for more than half of the world's population. Notably, scientists in China have developed several types of rice that can be grown in seawater, avoiding the use of precious freshwater resources and potentially creating enough food for 200 million people. The plant-affecting fungus Magnaporthe grisea is the causal agent of rice blast disease, and biological rather than chemical control of this threatening disease is highly desirable. In this work, we discovered fengycin BS155, a cyclic lipopeptide material produced by the marine bacterium Bacillus subtilis BS155, which showed strong activity against M. grisea. Our results elucidate the mechanism of fengycin BS155-mediated M. grisea growth inhibition and highlight the potential of B. subtilis BS155 as a biocontrol agent against M. grisea in rice cultivation under both fresh- and saltwater conditions.

## INTRODUCTION

The phytopathogen Magnaporthe grisea can cause serious disease in many species of the grass family, including economically important crops such as rice, wheat, and barley (1). In particular, rice blast disease caused by M. grisea is extremely difficult to control, and outbreaks of this disease could lead to significant economic and humanitarian problems (2, 3). While chemical control agents are still vital for the effective control of fungal plant pathogens and rice blast, the excessive use of chemicals increases the potential for the buildup of resistance in M. grisea, generating pathogen strains that are harder to control (4). Moreover, with the public concern over the hazardous effects of chemicals, it is very important to find and develop suitable alternatives for plant disease control (5). Biological control agents (BCAs), including microorganisms, microbial products, and biofertilizers, have been developed as promising and ecofriendly alternatives to chemical pesticides (5).

Over the past few decades, hundreds of peptide antibiotics have been described, and antimicrobial peptides sourced from microorganisms are considered promising drug candidates for the future because of their broad range of activity, reduced toxicity, the potential for environmentally friendly mass production, and the decreased resistance development associated with their use (6). Bacillus species synthesize a range of cyclic lipopeptides (CLPs) with broad-spectrum antimicrobial properties (7). These CLPs, consisting of a fatty acyl chain bound to a cyclic peptide ring, are produced by multidomain enzymes called nonribosomal peptide synthetases (NRPSs) (8). CLPs from Bacillus species can be divided into three main subfamilies, surfactins, iturins, and fengycins or plipastatins. Among these CLPs, fengycins exhibit powerful antifungal activity against a wide range of phytopathogens (9). Fengycin homologues effectively inhibit the growth of filamentous fungi, such as Rhizopus stolonifer, Gibberella zeae, Aspergillus niger, Mucor rouxii, Fusarium graminearum, and Sclerotinia sclerotiorum (10–13). Fengycin and the closely related plipastatin CLPs are composed of an N-terminal β-hydroxy fatty acid chain attached to a decapeptide forming a cyclic lactone ring. The major members of the fengycin subfamily are fengycin A and fengycin B, which differ structurally only by the residue at position 6 being Ala or Val, respectively (9). Further heterogeneity among the fengycins is introduced by the variable length of the β-hydroxy fatty acid chains (9).

Much work has been carried out to understand the molecular mechanisms of the biological activity of fengycins (14–17). Due to their amphiphilic nature, fengycins are believed to induce cell death by interacting with the cell membrane and increasing cell permeability (18, 19). Fengycins were shown to cause ultrastructural destruction of the fungal pathogen hyphae; fengycin-treated hyphae exhibited unconsolidated cytoplasm and cell walls that were gapped and/or separated from the cell membrane (14, 15). Reactive oxygen species (ROS) can oxidize lipids, proteins, DNA, and carbohydrates within biological organisms, leading to the breakdown of the cellular membrane or cell death (20). Recent research showed that CLPs, including bacillomycin D, iturins, and fengycins, were involved in an antagonistic interaction with the plant fungal pathogens by inducing ROS production (10, 21, 22). Additionally, certain CLPs specifically affect the signaling pathways of fungal or cancer cells. For example, bacillomycin D and iturins induce Hog1 mitogen-activated protein kinase (MAPK) activation and subsequent defects in the cell wall integrity (CWI) (21, 22). Surfactins were able to cause cell death of human breast cancer MCF-7 cells through the ROS/c-Jun N-terminal kinase-mediated mitochondrial/caspase pathway (23). Another study revealed that surfactins induce a collapse of mitochondrial membrane potential (MMP), initiating the release of cytochrome *c* from mitochondria and the activation of caspase 9, in MCF-7 cells (24). However, there are only few examples of investigations of the signaling pathways and intracellular cell responses to fengycins.

Presently, microbial proteomic technologies not only provide a powerful tool to profile protein expression in response to various stresses in a microorganism but also are used to reveal the possible mechanism of action of antibiotics (25–27). For instance, comparative proteomic analyses revealed that bacillomycin L interacts with intracellular targets of Rhizoctonia solani hyphal cells and affects mitochondrial function, RNA processing and silencing, and calcium homeostasis (28). Until now, proteomics-based mechanism studies of the action of fengycins against phytopathogens have not been reported.

Although many fengycin CLPs produced by Bacillus species have antiphytopathogen activities, their exact mechanism of action remains poorly understood. In the present study, fengycin BS155 from the marine bacterium Bacillus subtilis BS155 was shown to possess strong fungicidal activity against M. grisea. In this fungus, fengycin BS155 induced ROS production and chromatin condensation and, ultimately, caused cell death. These findings shed new light on antifungal mechanisms of fengycins and provide a new BCA against the notorious phytopathogen M. grisea.

## RESULTS

### Antifungal activity of marine bacterium B. subtilis BS155 against M. grisea.

To obtain potential antifungal agents, more than 400 marine bacteria were screened and evaluated on their ability to inhibit M. grisea growth by using an antagonistic experiment. Among these, strain BS155 showed the strongest activity against M. grisea (see Fig. S1A in the supplemental material). The diameter of the hyphal colony of M. grisea treated with BS155 crude extract (40 μg/ml) was only 2.25 cm, much smaller than that of the control untreated colony (6.65 cm) (Fig. S1B and C). According to the high homology (99% identity) with Bacillus subtilis strain 168, confirmed by 16S rRNA gene sequencing and phylogenetic tree analysis (see Fig. S2), the bacterium strain BS155 was designated Bacillus subtilis BS155.

### Purification, structure elucidation, and encoding gene determination of CLPs derived from B. subtilis BS155.

**FIG 1:**
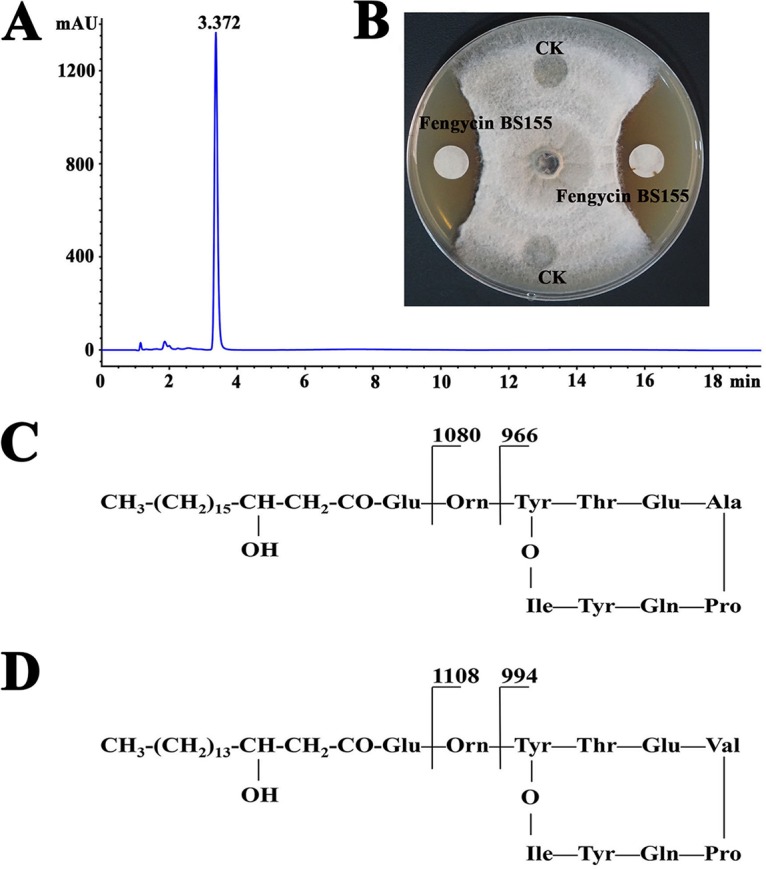
Purification and characterization of the active compounds responsible for M. grisea growth inhibition by B. subtilis BS155. (A) HPLC chromatogram of the active compound(s) from B. subtilis BS155. (B) Growth inhibition of M. grisea hyphal colonies by the active compound(s) from B. subtilis BS155. Chemical structures and fragmentation patterns of C_19_-fengycin A (C) and C_17_-fengycin B (D), which were isolated from B. subtilis BS155 as the active compounds.

To obtain the active compound(s) from B. subtilis BS155 responsible for M. grisea growth inhibition, purification was performed as described in Materials and Methods. The filter paper disc assay was carried out to follow the trail of the bioactive compound(s) using M. grisea as the indicator fungus. Notably, a single peak associated with strong antifungal activity was observed in the reversed-phase high-performance liquid chromatography (RP-HPLC) chromatogram (Fig. 1A), indicating that there was only one or a few closely related compounds responsible for the action of B. subtilis BS155. In the filter paper disc assay, the compounds collected from RP-HPLC showed strongly antifungal activity against M. grisea (Fig. 1B) and were further analyzed with a mass spectrometer to elucidate the chemical structure. The data of electrospray ionization mass spectrometry (ESI-MS) and tandem mass spectrometry (ESI-MS/MS) showed that the ions corresponding to the active compound(s) were [M + Na]^+^ (*m/z* 1,527.8134) and [M + H]^+^ (*m/z* 1,505.8307) (see Fig. S3A). Thus, the molecular weight of the purified active compound(s) was determined to be 1,504 Da. Notably, these results corresponded to the MS data obtained from fengycin CLPs in previous reports (16, 29–31). The profile of the typical fragmentation ions of *m/z* 966 and 1,080 in the ESI-MS/MS spectrum can be explained by neutral losses of fatty acid-Glu and fatty acid-Glu-Orn, respectively, from the N-terminal segment with an Ala residue at position 6 in C_19_-fengycin A cyclic decapeptide. In addition, the neutral losses of fatty acid-Glu and fatty acid-Glu-Orn from the N-terminal segment with a Val residue at position 6 in C_17_-fengycin B cyclic decapeptide were attributed to the fragmentation ions *m/z* 994 and 1,108, respectively (Fig. S3B) (32, 33). C_19_-fengycin A and C_17_-fengycin B had β-hydroxy fatty acids with chain lengths of 19 and 17 carbon atoms, respectively, leading to a mass difference of 28 Da. However, Ala (71 Da) and Val (99 Da) residues appeared at position 6 of the cyclic decapeptides in C_19_-fengycin A and C_17_-fengycin B, respectively, which counteracted the 28-Da mass difference resulting from different β-hydroxy fatty acid chain lengths. The pairs of typical fragments (*m/z* 966 and 1,080 and *m/z* 994 and 1,108) were present simultaneously and led us to conclude that both C_19_-fengycin A and C_17_-fengycin B contributed to the production of the ion of *m/z* 1,505.8307 (9, 34). Overall, the data of the ESI-MS and ESI-MS/MS spectra are consistent with fengycin compounds, specifically with C_19_-fengycin A and C_17_-fengycin B CLPs (Fig. 1C and D) (17, 35). Thus, the active metabolites from B. subtilis BS155 were C_19_-fengycin A and C_17_-fengycin B, which have the same molecular weight and are collectively referred to as fengycin BS155 in this study.

To determine the potential genes that encode fengycin BS155 CLPs, the entire genome of B. subtilis was sequenced. The results indicated that the genome of B. subtilis BS155 consists of one circular chromosome of 4,325,794 bp with 43.49% GC content (see Table S1). It was reported that the gene clusters *fen* and *pps* were deemed to share the same five open reading frames of *ppsA* to *ppsE* or *fenA* to *fenE* (36). Notably, five genes were proposed to be involved in the synthesis of fengycin BS155 in B. subtilis BS155, i.e., *fenC*, *fenD*, *fenE*, *fenA*, and *fenB* (Fig. S4). Therefore, we named this gene cluster *fenA–E*, and it was localized in the single chromosome between nucleotide 2,171,908 and nucleotide 2,209,634. The sequence alignment results showed that the gene cluster *fenA–E* shared 97.91% similarity with the corresponding gene cluster in reference strain B. subtilis 168. Approximately 11 gene clusters encoding NRPSs and polyketide synthases (PKSs) were found in B. subtilis BS155. Among these, the PKS gene cluster 7 shared a low level of homology with corresponding gene clusters in other Bacillus species. Interestingly, the *sfp* gene cluster encoding surfactin was also found in B. subtilis BS155, and the *sfp* gene plays an important role in the induction of B. subtilis 168 to produce plipastatin or fengycins (37).

### Ultrastructural morphology and viability changes of M. grisea hyphal cells caused by fengycin BS155.

**FIG 2:**
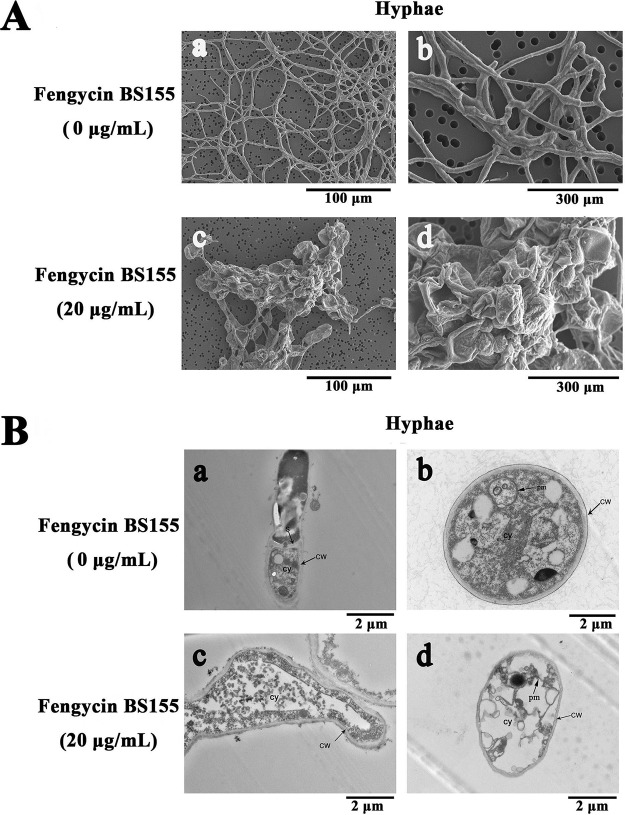
Effect of fengycin BS155 on the morphology and ultrastructure of M. grisea hyphae, observed by SEM (A) and TEM (B). In the control groups, M. grisea hyphal cells were treated with methanol (0.04% [vol/vol]) (a and b in panels A and B). In the test groups, M. grisea hyphal cells were treated with 20 μg/ml fengycin BS155 (c and d in panels A and B). CW, cell walls; cy, cytoplasm; pm, plasma membrane; S, septum.

To study the effects of fengycin BS155 on the morphology and ultrastructure of M. grisea hyphae, scanning electron microscopy (SEM) and transmission electron microscopy (TEM) were conducted. When the hyphal cells of M. grisea were treated with fengycin BS155 at a concentration of 20 μg/ml for 48 h, the resulting morphological variations were first checked by SEM. The results showed that the untreated hyphae of M. grisea had regular, plump, and intact trunks (Fig. 2A, a and b). On the contrary, the morphology of hyphae treated with fengycin BS155 was obviously different from that of the normal control hyphae. Unlike the slender hyphae of the control group, the fengycin BS155-treated hyphae appeared swollen, coarse, and irregular, with distorted grooves and corrugations (Fig. 2A, c and d). Then, TEM was used to observe the effects of fengycin BS155 on the ultrastructure of M. grisea hyphae. The normal fungal hyphal cells had intact and regular cell walls and cell membranes and typical septa and plasma membranes (Fig. 2B, a and b), and the dense cytoplasm was uniformly distributed in the intracellular space. However, the cell membrane became incomplete and the well-distributed cytoplasmic content became sparse and light, even partially lost, after fengycin BS155 treatment (Fig. 2B, c and d). Altogether, the electronic microscopy (EM) results suggested that the mechanism of action of fengycin BS155 against M. grisea was associated with damage to the cytoplasm, cell and plasma membranes, and cell integrity.

**FIG 3:**
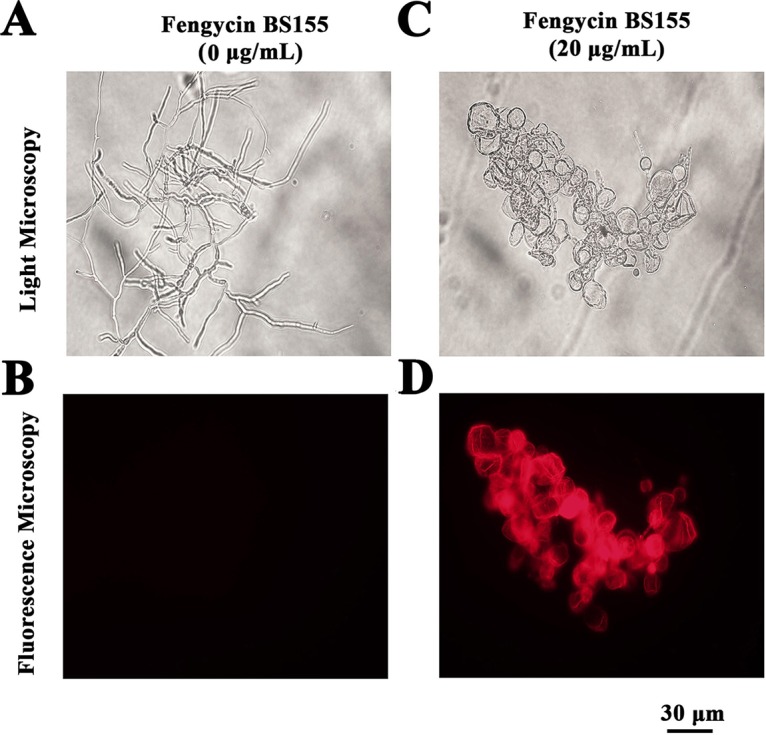
Effect of fengycin BS155 on viability and membrane integrity in M. grisea hyphal cells, observed by light microscopy (A and C) and fluorescence microscopy (B and D). Live fungal hyphal cells with intact membranes show no fluorescence under fluorescence microscopy (B); the damaged cell membranes of fungal hyphae showed red fluorescence (D). Methanol (0.04% [vol/vol]) served as the control treatment, and the scale bar applies to all panels.

Next, hyphal cell viability and cell membrane integrity of M. grisea were examined by propidium iodide (PI) staining. The results showed that the fengycin BS155-treated hyphae displayed much stronger red fluorescence than the control, which clearly indicated that the CLPs triggered fungal hyphal cell membrane defects and cell death (Fig. 3). These viability results were consistent with the EM observations described above (Fig. 2).

### Accumulation of ROS in M. grisea hyphae induced by fengycin BS155.

**FIG 4:**
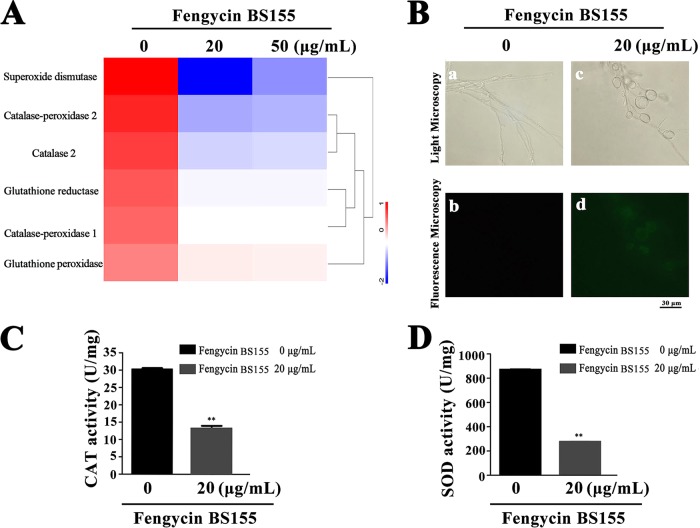
Reactive oxygen species (ROS) accumulation in M. grisea hyphal cells after treatment with fengycin BS155. (A) Proteomic, clustering, and heatmap analyses of differentially expressed ROS-scavenging enzymes following treatment with different concentrations of fengycin BS155. The relative protein expression levels of ROS-scavenging enzymes (fold change ≥1.5 or fold change ≤0.67 and *P* < 0.05) were clustered and analyzed. (B) ROS accumulation of M. grisea hyphal cells observed by light microscopy (a, c) and fluorescence microscopy (b, d). Representative fluorescence micrographs of M. grisea hyphal cells stained with 10 μM 2′,7′-dichlorofluorescein diacetate after treatment with 0 μg/ml (b) or 20 μg/ml (d) fengycin BS155. The scale bar applies to all panels. Effects of fengycin BS155 on the activities of CAT (C) and SOD (D), the ROS-scavenging enzymes, in M. grisea hyphae. The fungal hyphal cells were treated with 0 μg/ml or 20 μg/ml fengycin BS155 prior to enzyme activity assays. The mean expression values ± SDs are reported relative to the control. **, *P* < 0.01.

To better describe the mechanism of the antifungal activity of fengycin BS155 against M. grisea, a proteomic assay was performed. The results of this assay showed that approximately 1,211 and 1,258 proteins were expressed differentially (>1.5-fold or <0.667-fold change and *P* < 0.05) relative to the control after the treatment of M. grisea hyphae with 20 μg/ml and 50 μg/ml fengycin BS155, respectively. The differentially expressed proteins were mainly localized to cellular organelles, membrane, and membrane-enclosed lumen (see Fig. S5A and B). Furthermore, an analysis of the distribution and subcellular location of the differentially expressed proteins revealed that the proteins of the nucleus, cytoplasm, mitochondria, plasma membrane, and cytoskeleton undergo a significant change in abundance upon treatment with fengycin BS155 (Fig. S5C and D), implying that these hyphal subcellular structures were impacted by the CLPs. Notably, among all the downregulated proteins, six types of ROS-scavenging enzymes, i.e., superoxide dismutase (SOD; MGG_07697), catalase (CAT; MGG_10061), catalase-peroxidase 1 (KATG1), catalase-peroxidase 2 (KATG2), glutathione reductase (MGG_12749) (38, 39), and glutathione peroxidase (MGG_07460), were significantly depleted (Fig. 4A). As unavoidable byproducts of aerobic metabolism, ROS play important physiological roles, whereas an excess of these species has the capacity to damage cell components and induce cell death (40). Several lipopeptides have been reported to induce the burst of ROS in certain fungal pathogens (21, 22). To clarify whether M. grisea cells accumulate ROS after fengycin BS155 treatment, a ROS detection assay, based on the fluorescence of 2′,7′-dichlorofluorescein diacetate (DCFH_2_-DA) staining, was applied. Indeed, the fengycin BS155-treated hyphae of M. grisea showed strong green fluorescence (Fig. 4B, c and d), while the untreated control hyphae had almost no green fluorescence (Fig. 4B, b). In particular, the fluorescence intensity originated mainly from the distorted and swollen hyphal cells (Fig. 4B, d). These observations demonstrated that fengycin BS155 induces the accumulation of ROS in the hyphae. Given that ROS-scavenging enzymes were significantly downregulated according to our proteomic results, we proposed that these enzymes might contribute to the accumulation of ROS in fengycin BS155-affected hyphae of M. grisea.

To further verify the downregulation of ROS-scavenging enzymes demonstrated by the proteomic assay, the activities of CAT and SOD in M. grisea hyphal cells were measured with or without fengycin BS155 treatment. The CAT enzyme activity in hyphal cells treated with 20 and 50 μg/ml of fengycin BS155 was 13.16 and 10.00 U/mg, respectively, and was strongly reduced compared to that of the control group (30.20 U/mg) (Fig. 4C). These results indicated that the CAT activity was dramatically decreased by fengycin BS155 treatment (*P* < 0.01). The SOD enzyme was also strongly inhibited by the CLPs. The activity of this enzyme in hyphal cells treated with 20 and 50 μg/ml fengycin BS155 was 275.73 and 158.49 U/mg, respectively, while that of the control group was as high as 869.06 U/mg (Fig. 4D). Thus, the SOD activity of hyphal cells treated with fengycin BS155 was dramatically decreased after fengycin treatment (*P* < 0.01). Notably, fengycin BS155 reduced the activities of CAT and SOD in a dose-dependent manner, which was consistent with the proteomic data.

**FIG 5:**
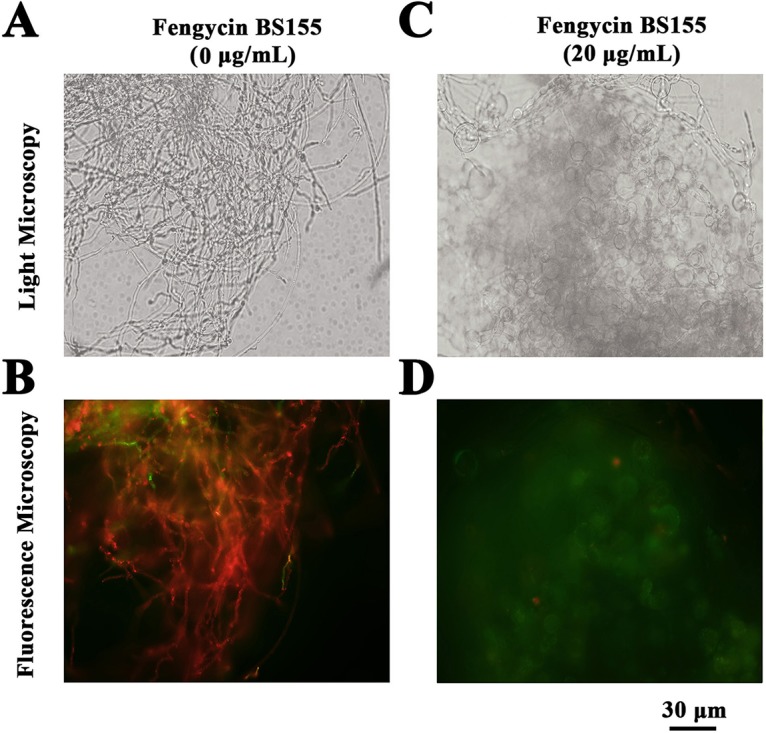
Effect of fengycin BS155 on mitochondrial membrane potential (MMP) in M. grisea hyphal cells, observed by light microscopy (A and C) and fluorescence microscopy (B and D). Representative fluorescence micrographs of M. grisea hyphal cells stained with JC-1 after treatment with 0 μg/ml (B) or 20 μg/ml (D) fengycin BS155. The scale bar applies to all panels.

It has been reported that ROS accumulation affects the MMP (41). Therefore, fluorescence microscopy was used to examine the changes in MMP in M. grisea hyphal cells after fengycin BS155 treatment. Indeed, the control group showed red fluorescence due to a high MMP, which resulted in aggregated JC-1 staining in the mitochondria (Fig. 5B), while the hyphal cells of M. grisea treated with fengycin BS155 displayed green fluorescence because of MMP reduction, which led to dispersed JC-1 staining in the mitochondria (Fig. 5D), demonstrating that the CLPs reduced the MMP in M. grisea hyphal cells.

### Induction of chromatin condensation by fengycin BS155 in M. grisea hyphae.

**FIG 6:**
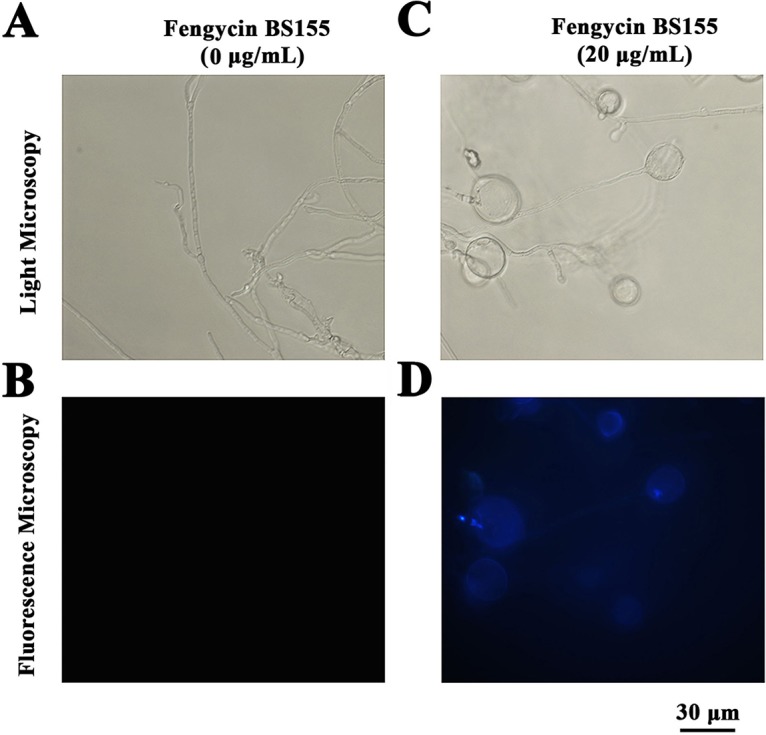
Fengycin BS155 induced chromatin condensation in M. grisea hyphal cells. Induction of chromatin condensation in M. grisea hyphal cells after fengycin BS155 treatment observed by light microscopy (A and C) and fluorescence microscopy (B and D). Representative fluorescence micrographs of M. grisea hyphal cells stained with Hoechst 33258 after treatment with 0 μg/ml (B) or 20 μg/ml (D) fengycin BS155. The scale bar applies to all panels.

During cell death, chromatin undergoes a phase change from a heterogeneous genetically active network to an inert highly condensed form (42). When stained with DNA-binding nuclear dyes, the compacted chromatin appears brighter than the chromatin in nonapoptotic cells and the condensed nuclei can be easily identified by fluorescence microscopy (10). To explore the effects of fengycin BS155 on the chromatin of M. grisea, Hoechst 33258 staining was performed. Under a fluorescence microscope, the hyphae not treated with fengycin BS155 exhibited faint blue fluorescence (Fig. 6B). On the contrary, the fengycin BS155-treated hyphae displayed strong blue fluorescence, indicative of chromatin condensation (Fig. 6D).

**FIG 7:**
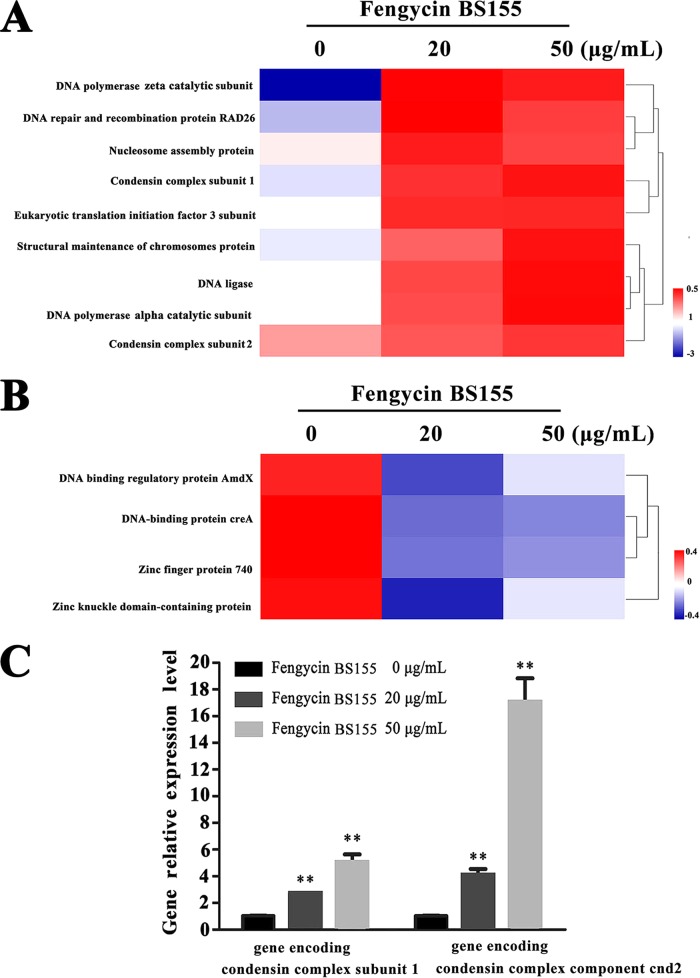
Proteomic, clustering, heatmap, and qRT-PCR analyses show the effect of fengycin BS155 treatment on chromatin condensation and DNA repair in M. grisea hyphal cells. (A) The relative levels of upregulation of chromatin condensation- and DNA repair-associated proteins (fold change ≥1.5 and *P* < 0.05) were clustered and analyzed. (B) The relative levels of downregulation of DNA stability maintenance proteins (fold change ≤0.67 and *P* < 0.05) were clustered and analyzed. (C) qRT-PCR assays of expression levels of genes encoding condensin complex subunit 1 (MGG_03487) and condensin complex component cnd2 (MGG_01639). The actin gene was used as an internal reference, and the data are represented as means ± SDs from three independent experiments. **, *P* < 0.01.

Nuclear chromatin condensation, along with concomitant DNA fragmentation, is one of the most important criteria used to identify apoptotic cells (10). Consistently, in the differentially expressed proteins quantified by our above-mentioned proteomic analysis, a total of 93 nucleus-associated proteins were identified, 16 of which were significantly altered at the level of their expression. Among these 16 proteins, 9 were markedly upregulated and implicated in chromatin condensation and DNA repair, i.e., DNA polymerase zeta catalytic subunit (MGG_02986), DNA repair and recombination protein RAD26 (MGG_05239), eukaryotic translation initiation factor 3 subunit (NIP1), condensin complex subunit 1 (MGG_03487), nucleosome assembly protein (MGG_06924), structural maintenance of chromosomes protein (MGG_07098), DNA ligase (MGG_06370), DNA polymerase alpha catalytic subunit (MGG_06397), and condensin complex component cnd2 (MGG_01639) (43–47) (Fig. 7A). Four proteins essential for maintaining DNA stability, DNA-binding regulatory protein AmdX (MGG_08757), DNA-binding protein creA (MGG_11201), zinc finger protein 740 (MGG_00504), and zinc knuckle domain-containing protein (MGG_05948) (48, 49), were downregulated significantly following treatment with fengycin BS155 (Fig. 7B). Moreover, the reverse transcription-quantitative PCR (qRT-PCR) results showed that the relative expression levels of the genes encoding condensin complex subunit 1 and condensin complex component cnd2 were 2.74-fold and 4.10-fold (20-μg/ml fengycin BS155 treatment), respectively, and 5.08-fold and 17.10-fold (50-μg/ml fengycin BS155 treatment), respectively, greater than those of the control (Fig. 7C). These results demonstrated that the relative expression of genes encoding condensin complex subunit 1 and condensin complex component cnd2 was significantly upregulated, in a dose-dependent manner, after treatment with fengycin BS155.

**FIG 8:**
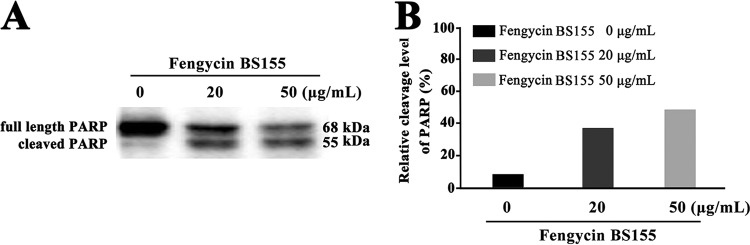
Induction of PARP cleavage by fengycin BS155 in M. grisea hyphal cells. (A) Analysis of PARP cleavage by Western blotting in the hyphal cells of M. grisea treated with fengycin BS155. (B) Quantification of PARP cleavage. The relative cleavage levels were calculated using Image lab.

The DNA repair enzyme poly(ADP-ribose) polymerase (PARP) can be activated by DNA strand breaks, and its cleavage is an important indication of cell death (43, 50). In this study, the Western blot analyses showed that in the control group, most PARP proteins were full length with a molecular weight approximately 68 kDa, and only a few PARP proteins were cleaved (Fig. 8A). On the other hand, the PARP enzymes were cleaved into truncated proteins with a molecular weight of approximately 55 kDa following the treatment with fengycin BS155 (Fig. 8A). A quantification analysis of the PARP cleavage rate was then performed. In the control group, this rate was only 8.62%, whereas in the treated groups, the cleavage rates reached 37.08 and 48.54% following treatment with 20 and 50 μg/ml fengycin BS155, respectively (Fig. 8B). Altogether, our results suggest that the inhibition of cell growth by fengycin BS155 was at least partly mediated by the induction of chromatin condensation and DNA damage, resulting in the overexpression of DNA repair proteins, the cleavage of PARP, and finally, cell death.

## DISCUSSION

Rice (Oryza sativa L.) is critical for human nutrition worldwide. As the world population is projected to increase to 9 billion by 2050, the world's rice production must increase by 25% or more to meet the demands imposed by this projected population growth. The fungus M. grisea is one of the most destructive plant pathogens and leads to outbreaks of rice blast disease, threatening global food security (3). BCAs based on Bacillus have proven to be effective in the management of a series of plant disease problems, particularly where high levels of disease resistance were observed (51). Members of the CLP family produced by Bacillus species have a well-recognized potential for controlling phytopathogen growth (36). In particular, the fengycin CLPs have exhibited strong inhibition activities against plant fungal pathogens such as Pythium ultimum, Botrytis cinerea, Gibberella zeae, and Sclerotinia sclerotiorum (13, 34, 52). In this study, the marine bacterium B. subtilis BS155 showed strong growth inhibition activity against M. grisea (see Fig. S1 in the supplemental material). The antifungal compounds were isolated, purified, and finally identified as CLPs C_19_-fengycin A and C_17_-fengycin B, collectively named fengycin BS155 (Fig. 1). Moreover, the fengycin BS155 synthesis gene cluster consisting of *fenA–E* genes was also identified in the genome of B. subtilis BS155 (Fig. S4).

Fengycins can induce cell death in many fungal pathogens, but the mechanisms of their antifungal activity remain unclear. The EM observations of M. grisea hyphal cells showed that many distorted grooves and ridges aggregated on the surfaces of swollen fungal mycelial (or hyphal) cells, indicating defects in cell wall integrity and cytoplasm content leakage following fengycin BS155 treatment (Fig. 2). It has indeed been reported that fengycins interact with lipid membranes and induce cytoplasmic content leakage (14, 18), which is consistent with our findings. The PI staining assay further confirmed that cell wall integrity defects and ultimately death of these swollen hyphal cells were caused by fengycin BS155 (Fig. 3). Altogether, our results suggest that fengycin BS155 CLPs cause severe damage to the plasma membranes and alterations to the cell walls in M. grisea hyphae, resulting in cell death. Cell wall integrity (CWI) and mitogen-activated protein kinase (MAPK) signaling pathways are believed to contribute to the maintenance of the cell wall (21, 22). The CLPs iturin and bacillomycin D were reported to induce fungal pathogen death by interfering with one or more of these signaling pathways (21, 22). Similar phenomena, such as the swelling of hyphal cells and cell integrity defects, were observed when M. grisea was treated with fengycin BS155 in this study (Fig. 2 and 3). However, it remains unknown whether fengycin BS155 affects the MAPK or CWI signaling pathway; this needs to be investigated in more detail.

Furthermore, our study of the fengycin BS155 mechanism of action showed that these CLPs induced high ROS accumulation in M. grisea hyphae (Fig. 4B). ROS produced by NADPH oxidases play a key role in host defense and multicellular differentiation in fungi (53). While ROS at a low concentration are an important intracellular messenger in many molecular events, large amounts of ROS are associated with cell death (54). In recent studies, CLPs such as iturin, fengycins, and bacillomycin D were found to cause fungal cell death by inducing ROS accumulation (10, 21, 22). In this work, six types of ROS-scavenging enzymes whose expression levels were apparently downregulated after treatment with fengycin BS155 were identified by the proteomic analysis (Fig. 4A). Moreover, the activities of two typical ROS-scavenging enzymes, SOD and CAT, were dramatically reduced after the treatment with fengycin BS155 (Fig. 4C and D). Additionally, the downregulation of expressions and activities of ROS-scavenging enzymes weakened the ability of M. grisea to neutralize ROS, which promoted the accumulation of these species. However, we cannot eliminate the possibility that damage to the ROS-scavenging proteins prevents the removal of a normal amount of ROS, which needs to be verified by Western blot analyses in the future.

Furthermore, an excessive oxidative stress burden may trigger an MMP collapse, which in turn leads to the increase of ROS generation (55). Consistently, fengycin BS155 apparently induced a collapse of the MMP in M. grisea hyphal cells (Fig. 5), which might lead to the release of mitochondrial intermembrane proteins into the cytoplasm, triggering cellular fragmentation and DNA damage and eventually causing cell apoptosis (24, 50). Collectively, the induction of ROS bursts by fengycin BS155 is suggested to contribute to cell death in M. grisea; ROS accumulation was correlated with the downregulation of ROS-scavenging enzymes and MMP collapse.

Notably, we observed that chromatin condensation was induced by fengycin BS155 in M. grisea hyphal cells (Fig. 6). In addition, chromatin condensation can activate the DNA damage response (DDR) and is an integral part of the associated signaling pathway (56). The DDR occurs in the context of chromatin structure changes, whereby architectural features of chromatin contribute to DNA damage signaling and repair (56). Indeed, the expression of proteins implicated in DNA synthesis and repair was dramatically upregulated, according to our proteomic data (Fig. 7A), while the expression of proteins essential for DNA stability was suppressed (Fig. 7B). The transcription levels of genes encoding condensin complex subunit 1 and condensin complex component cnd2 were both significantly elevated in fengycin BS155-treated M. grisea hyphal cells (Fig. 7C).

Moreover, the cleavage of PARP, an important DNA repair enzyme, is regarded as the primary representative biomorphic clue for apoptosis (57, 58). PARP can be cleaved from a full-length protein with molecular weight of 116 kDa to an 85-kDa fragment by caspase 3 during animal cell death (59). In Aspergillus nidulans, an 81-kDa or modified 85-kDa PARP protein [e.g., glycosylated or oligo(ADP-ribosylated)] can be cleaved to a 60-kDa fragment by caspase-like protein during sporulation (60). In this study, the 68-kDa full-length PARP was cleaved to a 55-kDa truncated protein when treated with fengycin BS155 (Fig. 8A), and the cleavage rate was dose dependent (Fig. 8B). Overall, fengycin BS155 CLPs can induce chromatin condensation which thereafter activates the DDR; the DDR, in turn, triggers multiple cellular events, including the activation of DNA repair pathways, arrest of the cell cycle to allow time for repair, and, in certain cases, the initiation of senescence or apoptosis programs (42).

**FIG 9:**
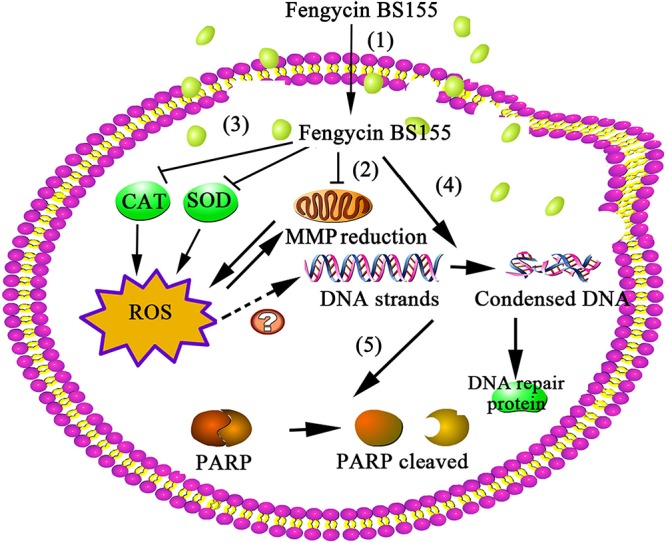
The proposed model of fengycin BS155 action in M. grisea hyphal cells. Fengycin BS155 interacts with the fungal hyphal cell membrane and enters the intracellular space (step 1). Fengycin BS155 damages the plasma membranes and then induces dysfunction of organelles and a burst of ROS (step 2). Fengycin BS155 reduces the MMP and downregulates the activities of ROS-scavenging enzymes (such as CAT and SOD), which in turn facilitates the production of additional ROS (step 3). Fengycin BS155 also induces DNA condensation, leading to the upregulation of DNA repair protein expression (step 4). Fengycin BS155 causes PARP cleavage (step 5), which is an important representative clue for hyphal cell apoptosis. Altogether, the organelle dysfunction, accumulation of ROS, and chromatin condensation induced by fengycin BS155 finally lead to cell death.

Altogether, the comparative proteomic and biochemical analyses of this study suggest that fengycins can induce high ROS accumulation and a collapse of the MMP and affect M. grisea DNA repair signaling pathways, leading to cell death (Fig. 9). Our study also highlights the potential of marine-derived bacterium B. subtilis BS155 as a BCA against M. grisea in rice cultivation under both fresh- and saltwater conditions.

## MATERIALS AND METHODS

### Microorganisms and culture conditions.

The marine bacterium BS155 used in this study was isolated from sediments collected near the Yap Trench during the seamount cruise of the R/V Kexue in the tropical Western Pacific in March 2016 (139°3802′E, 11°44162′N). The bacterium BS155 was maintained in modified Zobell 2216E broth (5 g tryptone and 1 g yeast extract in 1 liter filtered seawater, pH adjusted to 7.4 to 7.6) or Luria-Bertani (LB) medium (10 g/liter peptone, 5 g/liter yeast extract, 10 g/liter NaCl, pH adjusted to 7.0) and incubated at 28°C. The plant-pathogenic fungus M. grisea was purchased from Agricultural Culture Collection of China (accession number ACCC 37631) and maintained on potato dextrose broth (PDB) or agar (PDA) at 28°C for 5 days. To determine the phylogenetic position of bacterium strain BS155, PCR was performed to amplify the 16S rRNA gene sequence using the universal primers 27F (5′-AGAGTTTGATCCTGGCTCAG-3′) and 1541R (5′-AAGGAGGTGATCCACCC-3′). The 16S rRNA gene sequence was submitted to GenBank at the National Center for Biotechnology Information (NCBI) and compared with public databases by using the NCBI-BLAST and the phylogenetic analysis program (MEGA 6.0 software) (61).

### Screening of bacteria for inhibition of M. grisea growth.

To screen for bacteria able to inhibit the growth of M. grisea, cell suspensions of different bacteria were incubated overnight and adjusted to an optical density at 600 nm (OD_600_) of 0.2. The corresponding information about the fungus M. grisea can be found at the website http://www.accc.org.cn/Column_Content.asp?Column_ID=48571&pid=10160996. M. grisea was cultured on potato dextrose agar (PDA) plates at 28°C for 5 days. The mycelium plugs (diameter, 10 mm) taken from the edges of M. grisea cultures were placed at the centers of new plates. Ten microliters of different bacteria was seeded 3 cm away from the mycelium plug margin of M. grisea. The plates with M. grisea only, or with different bacteria and M. grisea, were incubated at 28°C for another 3 days before the fungal growth zone was measured.

### Isolation, purification, and identification of antifungal metabolites from B. subtilis BS155.

To identify the active compound(s) inhibiting the M. grisea growth, B. subtilis BS155 was cultured in a 250-ml glass flask filled with 2216E medium overnight at 28°C with shaking at 180 rpm. Then, 10 ml of this seed culture was transferred to a 3-liter flask containing 500 ml of LB medium. After incubation for another 48 h at 28°C, the bacterial cells were removed by centrifugation at 8,000 × *g* for 15 min. The pH of the supernatant was adjusted to 2 to 3 using a 6 N HCl solution and kept at 4°C overnight until the precipitation was complete. The precipitate was then collected by centrifugation at 8,000 × *g* at 4°C for 10 min and washed with 0.1 N HCl. Methanol was used to extract the precipitate. After the solvent was evaporated under vacuum using a rotary evaporator (at 40°C), the crude extract was concentrated and redissolved in methanol. The crude extract was loaded onto a Sephadex LH-20 column and eluted with methanol as the mobile phase. The collected fractions were used for assessing antifungal activity with the paper disc method as described previously; the M. grisea hyphal cells treated only with methanol (0.04% [vol/vol]) were selected as the control (62). The active metabolites were dissolved in methanol for RP-HPLC (Agilent 1260 Infinity; Agilent, USA) analyses using an Eclipse XDB-C_18_ column (5 μm, 150 mm by 4.6 mm) (Agilent, USA). A UV wavelength of 210 nm was used to detect product peaks with a flow rate of 1.0 ml/min with the mobile phase of methanol. The molecular weight data of the purified bioactive compounds were obtained by ESI-MS and ESI-MS/MS using a maXis mass spectrometer from Bruker, Germany. Data from ESI-MS were obtained under the following conditions: 3,200-V capillary voltage, 4.0-liter/min dry gas, and 200°C dry gas temperature. The multiple-reaction monitoring (MRM) mode was used to acquire ESI-MS/MS data, and all experiments were executed at 25°C. The antifungal activity of the purified compound with a final concentration of 40 μg/ml was tested, and the hyphal colony diameter was measured as described by Han et al. (21). Three independent assays were carried out, and the average values were calculated.

### Measuring M. grisea hyphal cell viability and cellular integrity.

Cell viability and cellular integrity were measured by PI staining, as described previously (10, 22). To produce M. grisea mycelium cells, the mycelium plug was fragmented from PDA medium and inoculated in 50 ml of CM broth (10 g glucose, 2 g peptone, 1 g yeast extract, 1 g Casamino Acids, nitrate salts, trace elements, 0.01% vitamins, and 1 liter water, pH 6.5). After incubation at 28°C for 24 h with shaking at 100 rpm, the culture was again inoculated in 50 ml of PDB (63). Fengycin BS155 preparations, dissolved in methanol, were injected into 50 ml of PDB to a final concentration of 20 μg/ml, and the resulting cultures were incubated for another 48 h. The methanol (0.04% [vol/vol]) treatment was used as the control. The collected fungal hyphal cells were resuspended with 10 mM phosphate-buffered saline (PBS), pH 7.2 to 7.4, and stained with PI at 28°C for 20 min in the dark. Thereafter, the hyphal cells were transferred to the microscope slides for microscopic observation using a filter (535 nm/615 nm) under a fluorescence microscope (Zeiss, Germany). Each assay was performed with three replicates.

### Ultrastructural changes of M. grisea hyphae following fengycin BS155 treatment.

SEM and TEM were used to investigate the morphological changes of M. grisea hyphae at the hyphal and ultrastructural levels after fengycin BS155 treatment. For SEM observation, the hyphal cells with or without fengycin BS155 treatment were collected and immersed in 10 mM PBS (pH 7.2 to 7.4) and filtered with 0.22-μm Nuclepore track-etched membranes (Whatman, England). The membranes containing hyphal cells were fixed in 5% glutaraldehyde for 1 h and dehydrated through 10-min washes in an ethanol gradient. All the samples were observed at 5 kV with SEM (S-3400N; Hitachi, Japan). For the TEM observations, the acquired fungal hyphal cells were prefixed with 2.5% glutaraldehyde. All the samples were examined and operated at 120 kV with TEM (HT7700; Hitachi, Japan).

### Protein sample preparation and proteomic data analyses.

For further analyses of the mechanism of action of fengycin BS155 against M. grisea, the proteomic assay and analyses were executed by PTM Biolab, Inc. (Hangzhou, Zhejiang, China). The M. grisea hyphal cells treated only with methanol (0.04% [vol/vol]) and with different concentrations of fengycin BS155 dissolved in methanol (20 or 50 μg/ml) were assigned as the control and experimental groups, respectively. Three parallel samples with methanol or fengycin BS155 treatment in the control or experimental groups were mixed uniformly and used for proteomic analyses. The hyphal cells were treated for 48 h with methanol or fengycin BS155 and collected, ground into powder using liquid nitrogen, and transferred to a 5-ml centrifuge tube. Total proteins of hyphal cells were digested, precipitated, and labeled. The obtained total protein samples were identified by liquid chromatography (LC)-electron spray ionization (ESI)-tandem mass spectrometry (MS/MS) analyses. For protein quantitation, a protein was required to contain at least two unique peptides. Protein quantitative ratios were weighted and normalized relative to the median ratio in Mascot as described by Kuang and colleagues (41). The collected raw data were further processed and pathway-based enrichment analyses of differentially expressed proteins were conducted with KEGG (Kyoto Encyclopedia of Genes and Genomes). For exported results, a changed protein that simultaneously met the conditions of two treatments (*P* < 0.05) and with fold changes of ≥1.5 or ≤0.66 was considered to be differentially expressed. The differentially expressed proteins were further analyzed using the software HemI (Heatmap Illustrator, version 1.0).

### MMP, ROS, and DNA condensation detection in fungal hyphae.

The effects of fengycin BS155 on the MMP were evaluated by fluorescence microscopy combined with JC-1 dye staining. M. grisea hyphal cells were treated with different concentrations of fengycin BS155 dissolved in methanol (0 μg/ml and 20 μg/ml) for 48 h and stained with JC-1 for 15 min at 28°C in the dark. These experiments were repeated by using three or more parallel samples. Fluorescence images of the treated cells were taken using a fluorescence microscope with a 515 nm/529 nm filter.

ROS induced by fengycin BS155 in M. grisea was detected according to a previous description (22). The analyses of hyphal cells treated with different concentrations of fengycin BS155 dissolved in methanol (0 μg/ml and 20 μg/ml) were conducted three times. Hyphal cells were treated with fengycin BS155 (0 μg/ml and 20 μg/ml) as described above. After treatment, the hyphal cells were stained with 10 μM 2′,7′-dichlorofluorescein diacetate (DCFH_2_-DA; Sigma-Aldrich) for 20 min at 28°C in the dark. The hyphal cells were observed and recorded under a fluorescence microscope with a filter (488 nm/525 nm) in the dark.

For the detection of DNA condensation, M. grisea hyphal cells treated with different concentrations of fengycin BS155 (0 μg/ml and 20 μg/ml) dissolved in methanol were collected, resuspended in 10 mM PBS (pH 7.2 to 7.4), and stained with 10 μg/ml Hoechst 33258 at 28°C for 20 min in the dark. The hyphal cells were investigated under a fluorescence microscope with a filter (346 nm/460 nm).

### CAT and SOD activities in M. grisea hyphal cells.

The total proteins of M. grisea hyphal cells were extracted as described previously (64). Briefly, the fungal hyphae were inoculated at 28°C for 48 h in 50 ml of liquid PDB medium containing different concentrations of fengycin BS155 (0 μg/ml and 20 μg/ml) dissolved in methanol. The treated hyphal cells were ground thoroughly using liquid nitrogen and a mortar and suspended in 10 mM PBS (pH 7.2 to 7.4). The sample was centrifuged at 8,000 × *g* for 10 min at 4°C, and the supernatant was transferred to a new tube. The protein concentration was measured using a bicinchoninic acid (BCA) protein assay kit according to the manufacturer's instructions (Solarbo, Beijing, China). CAT and SOD activities were checked with the corresponding assay kits (Jiancheng, Nanjing, China), and absorbance was measured with a spectrophotometer (Infinite M1000 Pro; Tecan, Männedorf, Switzerland), according to the manufacturer's instructions. Means and standard errors from three replicates per point are shown.

### qRT-PCR analyses.

qRT-PCRs were performed to analyze the relative expression levels of genes encoding condensin complex subunit 1 (MGG_03487) and condensin complex component cnd2 (MGG_01639), as previously described (28). Briefly, the hyphal cells were treated with fengycin BS155 dissolved in methanol at different concentrations (0 μg/ml, 20 μg/ml, and 50 μg/ml). Total RNA was extracted from the mycelia with TRIzol reagent and reversed transcribed into cDNA, which was used for subsequent PCR amplification. The primers for qRT-PCR were designed using Primer 5.0 and are listed in Table S2 in the supplemental material. Real-time PCR was performed using a SYBR green assay kit (Toyobo, Japan), with the QuanStudio 6 Flex real-time PCR detection system (Life Technologies, USA). The actin gene (MGG_03982.5) was employed as an internal reference (65). The relative expression levels of genes encoding condensin complex subunit 1 (MGG_03487) and condensin complex component cnd2 (MGG_01639) were calculated by using the ΔΔ*C_T_* method (66). All qRT-PCRs were conducted three times and the average values were analyzed.

### Western blot analyses.

Western blot analyses were executed as described by Gu et al. (22). Briefly, M. grisea hyphal cells were treated for 48 h with different concentrations of fengycin BS155 (0 μg/ml, 20 μg/ml, and 50 μg/ml) dissolved in methanol, and then approximately 200 mg of hyphal cells was collected and ground into powder with liquid nitrogen. The powder was resuspended into a buffer containing 50 mM Tris-HCl (pH 7.5), 100 mM NaCl, 5 mM EDTA, 1% Triton X-100, and 1% of protease inhibitor cocktail (Beyotime, China). The sample was centrifuged at 8,000 × *g* for 20 min at 4°C and the supernatant was collected. The concentration of total proteins therein was detected as described above. Approximately 24 μg total proteins was separated on a sodium dodecyl 10% polyacrylamide gel and transferred to nitrocellulose membranes. These membranes were then blocked for 2 h with 5% nonfat dried milk dissolved in Tris-buffered saline with Tween 20 (TBST; 150 mM NaCl, 10 mM Tris-HCl, 0.1% Tween 20, pH 7.5) and incubated overnight at room temperature with a primary antibody against PARP (Proteintech Group, Inc., China). After three washes with TBST, the membrane was incubated with an anti-rabbit horseradish peroxidase (HRP)-conjugated secondary antibody (Proteintech Group, Inc., China) at a 1:500 dilution. Antigen-antibody detection was performed with an enhanced chemiluminescence (ECL) solution. The integrated density values (IDVs) of bands were quantified by Image lab software.

### Statistical analyses.

All results were obtained from three independent experiments and are shown as the means ± standard deviations (SDs). The significant differences among groups were calculated with analyses of variance (ANOVAs) followed by Dunnett's tests by using GraphPad Prism 5 software (San Diego, CA, USA). *P* values of <0.05 were defined as statistically significant in our study.

### Accession number(s).

The GenBank accession number for the 16S rRNA gene of Bacillus subtilis BS155 is MG309549.1. The GenBank accession number for the whole genome of Bacillus subtilis BS155 is CP029052.

## Supplementary Material

Supplemental file 1
