# Supplementary material for: Fengycins, Cyclic Lipopeptides from Marine Bacillus subtilis Strains, Kill the Plant-Pathogenic Fungus Magnaporthe grisea by Inducing Reactive Oxygen Species Production and Chromatin Condensation
Source: Appl Environ Microbiol. 2018 Aug 31;84(18):e00445-18. doi: 10.1128/AEM.00445-18 (PMC6122000; doi:10.1128/AEM.00445-18)
Supplement: Supplemental file 1 [file zam018188718s1.pdf]

Supplementary information

Cyclic lipopeptides fengycins from marine bacterium *Bacillus subtilis* kill plant pathogenic fungus *Magnaporthe grisea* by inducing reactive oxygen species production and chromatin condensation

Linlin Zhang[a,b,c,d], Chaomin Sun[a,b,d*]

CAS Key Laboratory of Experimental Marine Biology, Institute of Oceanology, Chinese Academy of Sciences, Qingdao, 266071, China[a]; Laboratory for Marine Biology and Biotechnology, Qingdao National Laboratory for Marine Science and Technology, Qingdao, 266071, China[b]; University of Chinese Academy of Sciences, Beijing, 100049, China[c]. Center for Ocean Mega-Science, Chinese Academy of Sciences, Qingdao, 266071, China[d].

*Corresponding author

Chaomin Sun Tel.: +86 532 82898857; fax: +86 532 82898648.

E-mail address: sunchaomin@qdio.ac.cn

TABLE S1 General genome features of *B. subtilis* BS155

Features	Value
Genome size (bp)	4,325,794
G+C content (%)	43.49
Chromosome	1
Total number of genes	6,222
rRNAs	30
tRNAs	86
Genomic island	10
NRPS and PKS	11

TABLE S2 Oligonucleotides used in this study

Oligonucleotides	Sequence (5'-3')
Actin-se	AGCGTGGTATCCTCACTTTGC
Actin-an	ATCTGCGTCATCTTCTCTCGG
Conden1-se	ACATCTGCCTCGAGCAATACG
Conden1-an	AGCCTTGGGCTTCAGGAACA
Conden2-se	TGTGCGTGTCGGCAGAATACT
Conden2-an	CATGTCACCAAGGGCGATAAC

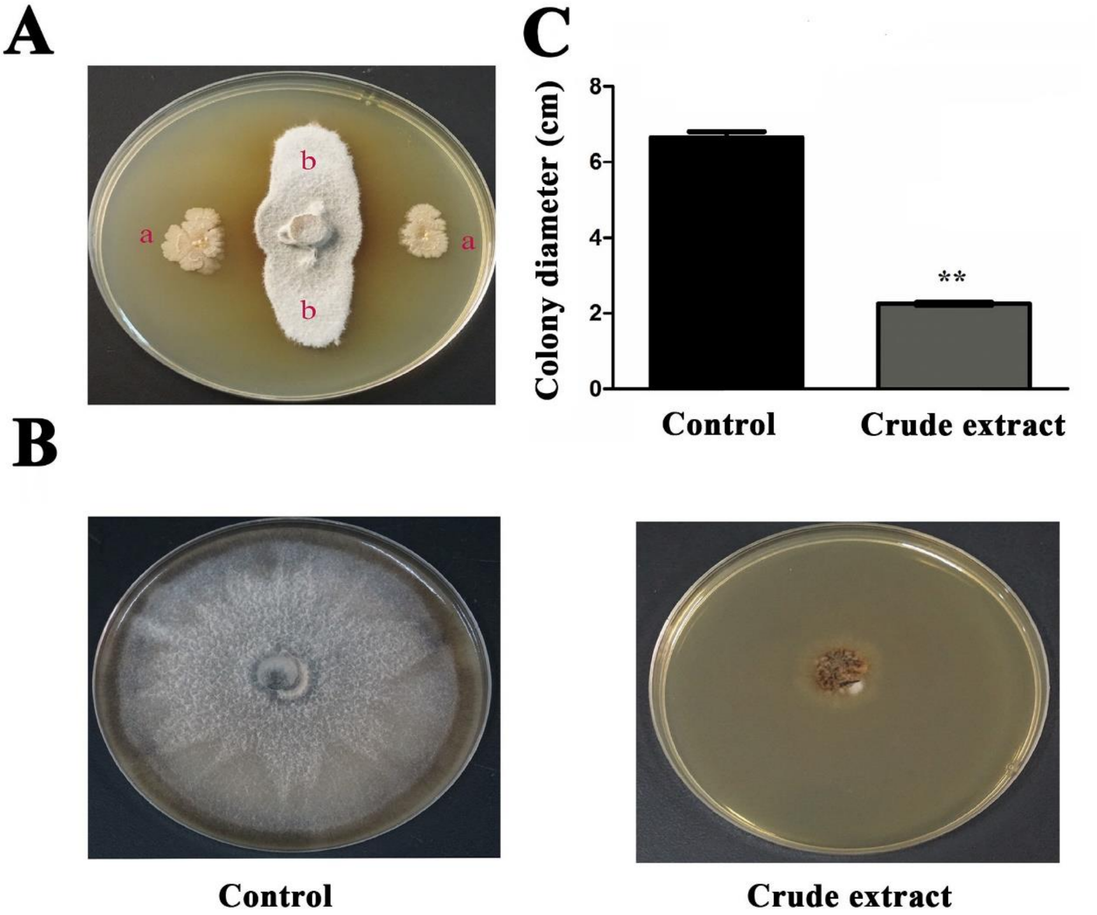

FIG S1 Antifungal activity of marine strain *B. subtilis* BS155 and its crude extract against *Magnaporthe grisea*. (A) Antagonistic activity of marine bacterium *B. subtilis* BS155 against *M. grisea*. a, strain *B. subtilis* BS155; b, fungal pathogen *M. grisea*. (B) Representative pictures of antagonistic activity assay of crude extracts (40 μg/mL) from *B. subtilis* BS155 against *M. grisea.* (C) Quantitative assays of antagonistic activity of crude extracts (40 μg/mL) from *B. subtilis* BS155 against *M. grisea*. **, $P < 0.01$.

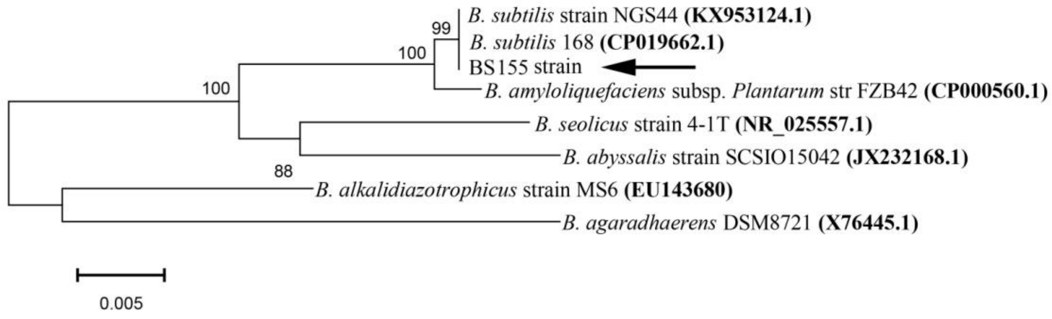

FIG S2 The consensus phylogenetic tree of marine bacterium BS155 with other related strains obtained from GenBank (accession numbers are indicated after the species name) constructed by the neighbor-joining method, respectively. Numbers above the branches are bootstrap values based on 1000 replicates.

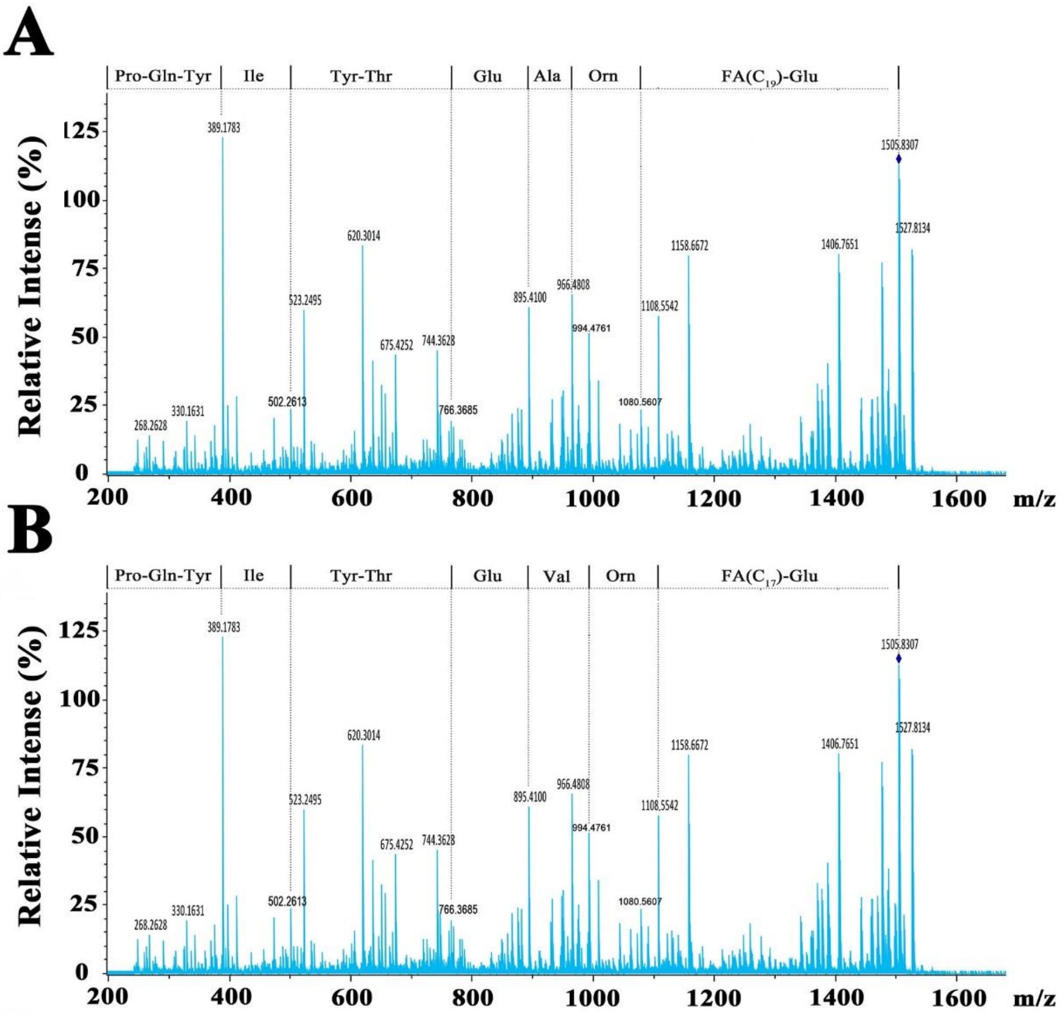

FIG S3 Fragmentation MS/MS spectrum of active compound peaks isolated from *B. subtilis* BS155. (A) The MS/MS spectrum of C_{19} fengycin A ion peaks from 200 Da to 1800 Da. (B) The MS/MS spectrum of C_{17} fengycin B ion peaks from 500 Da to 1500 Da.

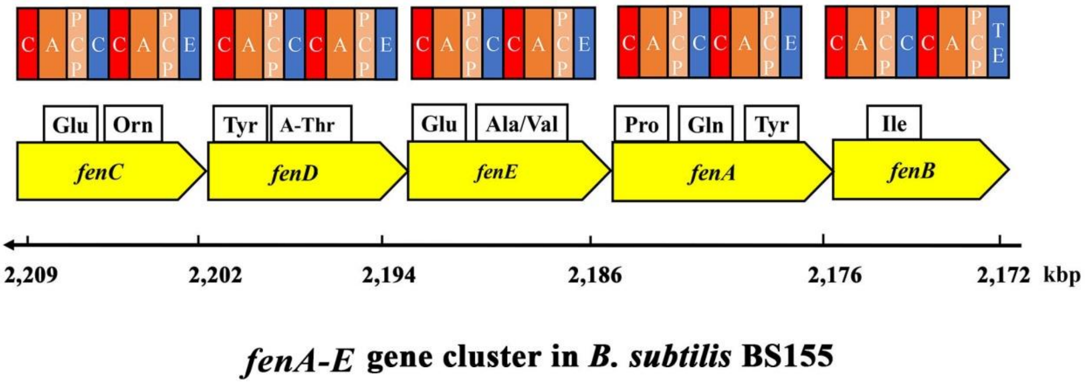

FIG S4 The predicted *fenA-E* gene cluster which was responsible for fengycin BS155 biosynthesis in the genome of *B. subtilis* BS155.

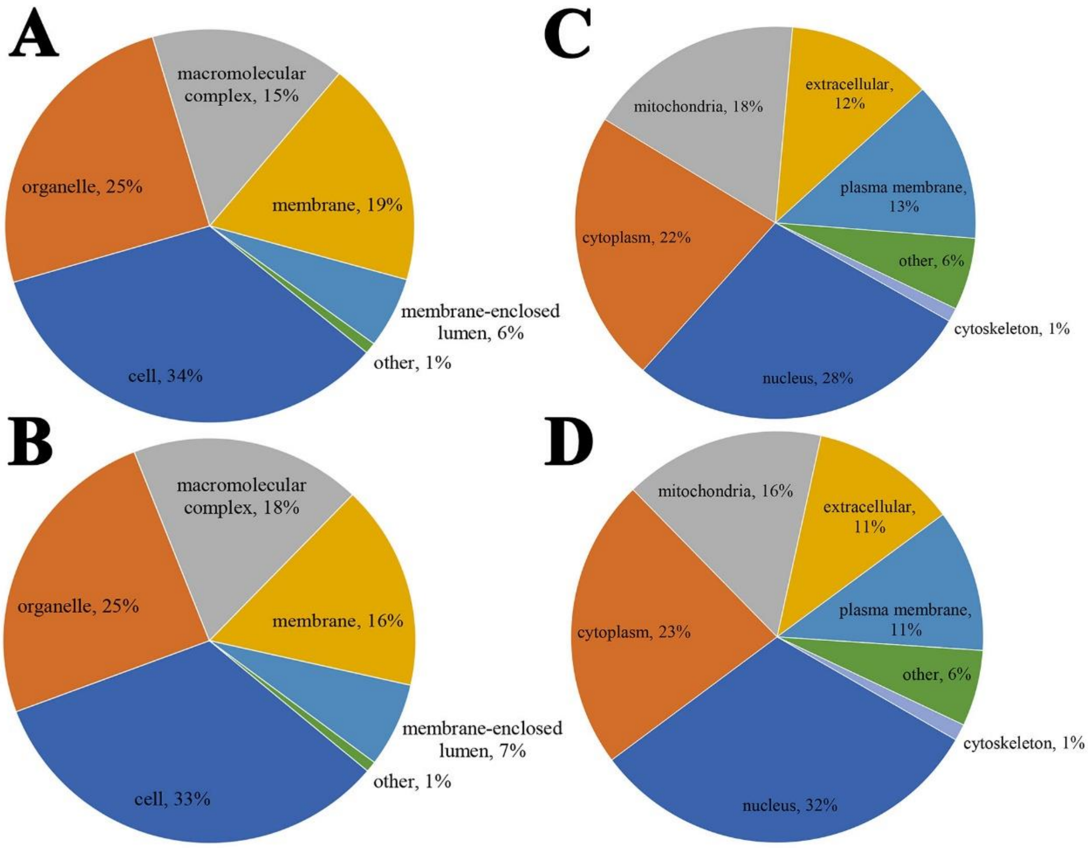

FIG S5 Hyphal cellular component cluster analyses of differentially expressed proteins according to the gene ontology annotation after *M. grisea* hypha was treated with 20 µg/ml (A) and 50 µg/ml fengycin BS155 (B). Subcellular localization analyses of differentially expressed proteins after the hyphal cells were treated with 20 µg/ml (C) and 50 µg/ml fengycin BS155 (D).
